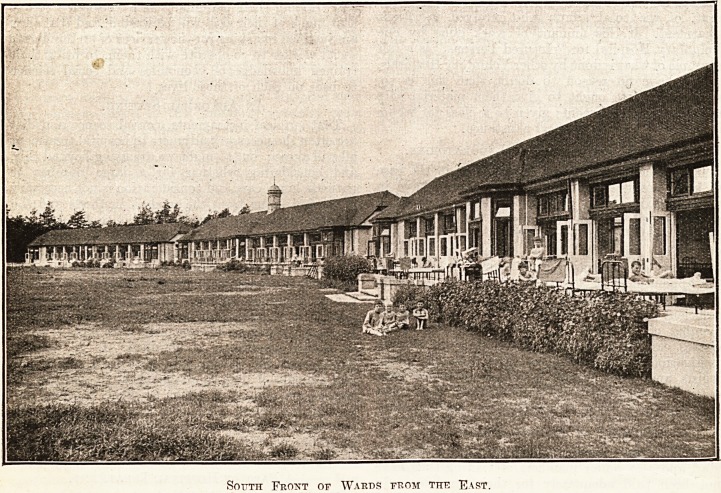# Heatherwood Hospital, Ascot

**Published:** 1923-07

**Authors:** 


### HEATHERWOOD HOSPITAL, ASCOT.

### HEALING BY NATURAL CONDITIONS.

HEATHERWOOD Hospital, Ascot, for the treatment of Ex-service men's children suffering from surgical tuberculosis, which was formally opened on May 29 by the Duke of Connaught, is a good exponent of the best modern methods of dealing with this serious and most common crippling condition among children. Originating in the efforts of the Army Children's Committee, and financed by the United Services Fund from the profits derived from the Expeditionary Force canteens, the work, though begun only in 1919, was so rapidly carried out that the hospital has been occupied by patients since June, 1922. Already good results are observed, though necessarily, many of the cases have to be under treatment for periods varying from a few months to two or even three years, and out of the 158 cases admitted, 29 of a mild type have been able to be discharged. The number of beds available, exclusive of 12 in the isolation block, is 138, which are distributed throughout three large wards each forming a self-contained block.

South Front of Wards from the East.

### Central Grouping.

The buildings are grouped together round a central garden court, the wards being connected by glass-covered ways. They all face south and the whole of the front of the wards open by folding doors on to a wide verandah for what is practically open-air treatment. One ward is for "babies" up to 7 years of age, one for girls and one for boys. The age limit is 14 years. Every facility for treatment is provided in the form of purposely constructed beds and cots, and highly specialised apparatus for the correction of various deformities; the Sister's room, in the centre of each ward, is provided with glazed walls, so that the little patients may readily be kept under constant observation. At one end of each ward are four cubicles each containing one cot where new patients are placed for the first 10 days or so of their stay in hospital as a preventive of possible infection, and at the other end of the centre ward is also a smaller one of six beds for any requiring special treatment.

### Conservative Treatment.

A covered way connects the wards with a Treatment Block containing large rooms for stores, plaster and splint work, an operating theatre, X-ray room, laboratory, dental room, &c., all thoroughly well equipped. The treatment carried out is on broad, conservative lines, both general and local, in which surgery does not occupy the sole or most prominent position. Great stress is laid on the healing powers of natural conditions to be found in a healthy climate, good hygiene and excellent dieting. The hospital possesses the advantage of being situated in a fine country on a sandy soil, where the climate is dry and bracing, the air clear, and the height above sea-level over 300 feet.

## Figures and Tables

**Figure f1:**